# The Relationship Between Social Support, Aging Expectations, and Health Behavior in Middle Age

**DOI:** 10.1093/geroni/igaa057.1317

**Published:** 2020-12-16

**Authors:** Hyun-E Yeom, Misook Jung, Eunyoung Park

**Affiliations:** 1 Chungnam National University, Daejeon, Taejon-jikhalsi, Republic of Korea; 2 Chungnam National University, Daejeon, Republic of Korea

## Abstract

Midlife is a critical period when individuals need to actively engage in healthy behaviors for healthy aging. Although both social relations and attitudes toward aging are factors related to health behavior, little is known about their relationships based on age-related differences. The purposes of this study were to investigate the influence of social support affecting health behavior through expectations regarding aging and to examine how age affects the relationship. A cross-sectional study was conducted with data from 245 midlife Koreans (mean age= 51.5) collected by a self-administered survey. Data were analyzed using the PROCESS macro in SPSS. Social support was significantly related to expectations regarding aging (r= -.135, p=.034) and health behavior (r= .223, p<.000). There was age-related difference in the relationship between social support and expectations regarding aging (β= .007, p=.038), indicating that the relationship was much stronger in the younger group. In addition, the influence of social support on health behavior through expectations regarding aging was significant in relatively young middle-aged individuals. Our findings emphasize the importance of supportive social relationships, which could affect expectations regarding aging linked to health behavior, especially for young middle-aged individuals. It is necessary to develop psycho-cognitive programs to activate social interaction and to improve positive attitudes toward aging for more active engagement in health behaviors in midlife individuals.

